# Transcriptomic Landscapes of Immune Response and Axonal Regeneration by Integrative Analysis of Molecular Pathways and Interactive Networks Post-sciatic Nerve Transection

**DOI:** 10.3389/fnins.2018.00457

**Published:** 2018-07-09

**Authors:** Qi Guo, Hui Zhu, Hongkui Wang, Ping Zhang, Shengran Wang, Zhichao Sun, Shiying Li, Chengbin Xue, Xiaosong Gu, Shusen Cui

**Affiliations:** ^1^Department of Hand Surgery, China-Japan Union Hospital, Jilin University, Changchun, China; ^2^Key Laboratory of Neuroregeneration, Ministry of Education and Jiangsu Province, Co-innovation Center of Neuroregeneration, Nantong University, Nantong, China; ^3^Jiangsu Clinical Medicine Center of Tissue Engineering and Nerve Injury Repair, Research Center of Clinical Medicine, Affiliated Hospital of Nantong University, Nantong, China

**Keywords:** axonal regeneration, immune response, peripheral nerve injury, Ingenuity Pathway Analysis, sciatic nerve transection

## Abstract

Potential interaction between immune response and axonal regeneration has recently attracted much attention in peripheral nervous system (PNS). Previously, global mRNA expression changes in proximal nerve segments were profiled and merely focused on the differentially change of the key biological processes. To further uncover molecular mechanisms of peripheral nerve regeneration, here we focused on the interaction between immune response and axonal regeneration that associated with specific molecular pathways and interactive networks following sciatic nerve transection. To offer an outline of the specific molecular pathways elaborating axonal regeneration and immune response, and to figure out the molecular interaction between immune response and axonal regeneration post-sciatic nerve transection, we carried out comprehensive approaches, including gene expression profiling plus multi-level bioinformatics analysis and then further experimental validation. Alcam, Nrp1, Nrp2, Rac1, Creb1, and Runx3 were firstly considered as the key or hub genes of the protein-protein interaction (PPI) network in rat models of sciatic nerve transection, which are highly correlated with immune response and axonal regeneration. Our work provide a new way to figure out molecular mechanism of peripheral nerve regeneration and valuable resources to figure out the molecular courses which outline neural injury-induced micro-environmental variation to discover novel therapeutic targets for axonal regeneration.

## Introduction

In contrast to the limited regenerative potential of central nervous system (CNS), peripheral nervous system (PNS) owns their capabilities to regenerate autonomously. Successful axonal regeneration is mainly due to the inherent regenerative capacity of neurons and superior microenvironment (Webber and Zochodne, 2010). Peripheral nerve injury (PNI) such as transection, crush or Wallerian degeneration elicited by chemical insult, lead to axonal destruction and fragments clearance concerning immune response (Vargas and Barres, 2007). In the proximal nerve stump, Schwann cells (SCs) de-differentiate to support axonal regeneration (Rosenberg et al., 2014). Therefore, sprouting of axonal growth cones is promoted by both intrinsic and extrinsic factors, with which the original synaptic targets begin to reestablish functional connections (Isaacman-Beck et al., 2015).

Recently, the potential overlap between immune response and axonal regeneration has attracted much attention. Various molecules have been found to play dual or multiple roles in biological processes of immune response and axonal regeneration (Shastri et al., 2013; Xanthos and Sandkuhler, 2014). The immune cells infiltration was observed during axonal regeneration, causing transient set back of motor recovery following PNI. Furthermore, peripheral nerve regeneration can be speeded up by an efficient approach to modulate immune response. The PNI can activate both the innate and the acquired immune responses. In the first period post-injury, myelin debris is removed by SCs, which recruit phagocytes to the lesion site by releasing neurotrophic factors, cytokines, and chemokines. It is well known that macrophages improve tissue clearance. The infiltrating CD4 T cells sourced pro-inflammatory cytokines and B lymphocytes sourced antibodies potentiate the phagocytic capacity in macrophages (Bombeiro et al., 2016). Of note, the success or failure of the regeneration process has been interfered by the interact and overlap mechanisms of nervous and immune system after PNI (Gaudet et al., 2011; Rotshenker, 2011; Shastri et al., 2013; Xanthos and Sandkuhler, 2014). However, to obtain a global perspective of how these molecules, coupled with the bioprocesses and signaling pathways of immune response and axonal regeneration are deliberately orchestrated to guide the intrinsic regenerative programs post-PNI, more comprehensive investigations are still required.

Clarifying the mechanisms of peripheral nerve regeneration help us to provide effective treatment for patients suffering PNI. Therefore, the aim of our study is to provide an outline for clarifying the precise molecular mechanisms of both immune response and axonal regeneration post-PNI. Based on the importance of the immune response for nerve regeneration, comprehensive approaches as gene expression profiling followed by multi-level bioinformatics analysis and experimental validation were carried out to compare the performance of differentially expressed genes and build protein-protein interaction (PPI) networks for immune response and axonal regeneration in response to sciatic nerve transection.

## Materials and methods

### Animal and surgery

Adult Sprague-Dawley (SD) rats (male) with the weight of 220 g ± 20 g were provided by the Experimental Animal Center of Nantong University. They were randomly divided into 5 groups (9 rats for microarray and qPCR, 6 rats for immunostaining, 15 rats for each group). The approval number for the animal studies was totally 75. Anaesthetization were performed by an intraperitoneal injection of complex narcotics which include trichloroacetaldehyde monohydrate (85 mg/kg), magnesium sulfate (42 mg/kg), sodium pentobarbital (17 mg/kg). The sciatic nerve was exposed and transected near the central position of femur following an incision on the lateral side of the mid-thigh in the left hind limb, both nerve stumps were left without any interfere and the incision was then closed. This study was carried out in accordance with the recommendations of Jiangsu Province, China and the US National Institute of Health Guide for the Care and Use of Laboratory Animals. The protocol was approved by the Administration Committee of Experimental Animals (SYXK (Su) 2012-0031).

### Sample preparation and microarray

Samples were obtained according to the previous methods with some modification (Li et al., 2013). In brief, proximal sciatic nerve stumps (0.5 cm) were obtained at 1, 4, 7, and 14 days post-nerve transection as well as normal nerve segment was collected in the corresponding position, respectively. According to the manufacturer's instructions, total RNAs were extracted by using Trizol (Life technologies, Carlsbad, CA). Nanodrop ND1000 spectrophotometer (NanoDrop Technologies, Wilmington, DE) and Agilent Bioanalyzer 2100 (Agilent technologies, Santa Clara, CA) were applied to determine the RNA quality of each sample. The cRNA was hybridized via a Gene Expression Hybridization Kit (Agilent Technologies, Santa Clara, CA). Then the labeled cRNA hybridized to microarray slides under 60°C for 17 h with a gene expression hybridization kit (Agilent Technologies, Santa Clara, CA). Then, the arrays were washed using a Gene Expression Wash Buffer Kit (Agilent Technologies, Santa Clara, CA) before stabilization and dehydration was performed (Agilent Technologies). The slides were analyzed by an Agilent Microarray Scanner (Agilent Technologies) and the raw data compiled with Agilent feature extraction software. National Engineering Center for Biochip at Shanghai (China) helped us to execute all steps from RNA amplification to the final scanner output. The mixed samples were obtained from three independent animals for each group. Each group included three biological replicates. In total, 15 microarrays were used in this study.

### Bioinformatics analysis

According to the previous works (Mingueneau et al., 2013; Li et al., 2015), principal component analysis (PCA), Euclidian distance calculation and hierarchical clustering were performed on log^2^-transformed mean-centered datasets filtered for expression values greater than 128 in any subsets and including only 10% of probes that showed highest variation across analyzed populations. PCA analysis was carried out by the “Population PCA” tool. (http://cbdm.hms.harvard.edu/LabMembersPges/SD.html). Euclidian distance calculation and hierarchical clustering was performed with the multi-experiment viewer (MeV 4.9, http://www.tm4.org/). The average expression profiles of differentially expressed genes was based on a Mann–Whitney *U*-test. *Z*-score normalized expression data were then calculated for specific biological processes. The average expression of the three duplicate probe sets was calculated at each time point (Viader et al., 2011; Li et al., 2013). The Venn diagrams were done using Venny 2.1.0 online tool (Perisic et al., 2016). The dynamic expression level of the selected genes was clustered in the heatmap using GENE-E analysis platform (https://software.broadinstitute.org/GENE-E/). Pathway analysis and PPI network of the expression data was done by Ingenuity Pathway Analysis (IPA, QIAGEN, Redwood City) for genes with fold change (FC) ± 2.0. *Z*-score was calculated by *z-score* = (*N*_+upregulated_ – *N*_−downregulated_)/${\sqrt{N}}_{\text{total}}$. The overall predicted activation state of the canonical pathways is reflected by the sign of the calculated *z*-score (<0: inhibited, >0: activated). Practically, as greater than 2 or smaller than −2, *z*-scores can be considered significant.

In the present work, the methods may effectively reduce interference by false positive results to improve repeatability and ensure the further validation with a higher success rate than that of retaining all the changeable probes. However, the probes with less significantly changes in expression and lower signal values than the background or control probes will be omitted undoubtedly. Those probes with expression values less than 128 in any subsets were considered relatively unreliable, which qPCR were previously carried out with rare validation. Therefore, to obtain the reliable and repeatable results, here we only focused on the probes with the expression values greater than 128 in any subsets and including only 10% of that showed highest variation across analyzed populations.

### Quantitative real time PCR (qPCR)

Prime-Script RT reagent Kit (TaKaRa, Dalian, China) and SYBR Premix Ex Taq (TaKaRa, Dalian, China) were applied in synthesizing reverse-transcribed complementary DNA and the subsequent PCR, respectively. The relative expression level of the selected genes was calculated using the comparative 2^−ΔΔ^Ct method. The sequences of primer pairs are provided in Supplementary Table 3. Pearson's correlation analysis was carried to compare qRT-PCR and microarray data.

### Histological validation

At 1, 4, 7, and 14 days post-surgery, nerve samples from proximal nerve stump and normal nerve sections (0 day) were obtained for cutting into longitudinal sections that were subjected to immunoffuorescent triple-staining with rabbit anti-ALCAM polyclonal antibody (1:100 dilution, Abcam), anti-CREB1 (1:800 dilution, CST), anti-NRP1 (1:200 dilution, Abcam), anti-NRP2 (1:100 dilution, CST), anti-RAC1 (1:100 dilution, Sigma), anti-RUNX3 (1:600 dilution, Abcam), mouse anti-NF200 (1:400 dilution, Sigma) and Hoechst 33342 (1:5,000 dilution, Life Technologies) respectively. Primary antibodies incubated with the nerve sections at 4°C overnight, followed by further reaction with the secondary antibody (Goat anti-Mouse IgG-Alex-488, 1:500 and Donkey anti-Rabbit IgG-Cy3, 1: 1,000) at 4°C overnight, and nerve sections were observed by the aid of a confocal laser scanning microscope (TCS SP2, Leica).

### Statistical analysis

For statistical analysis, the data were replicated in at least three experiments. Data are presented as means ± SEM. Multiple comparisons were performed with one-way ANOVA plus Bonferroni *post-hoc t*-test. The statistical analysis was carried out using SPSS Statistics 22.0 software package (IBM, Chicago, Illinois). Differences were considered significant at ^*^p-value < 0.05, ^**^p-value < 0.01.

## Results

### Overview of transcriptional change in rat proximal nerve stump following sciatic nerve transection

**Figure 1:**
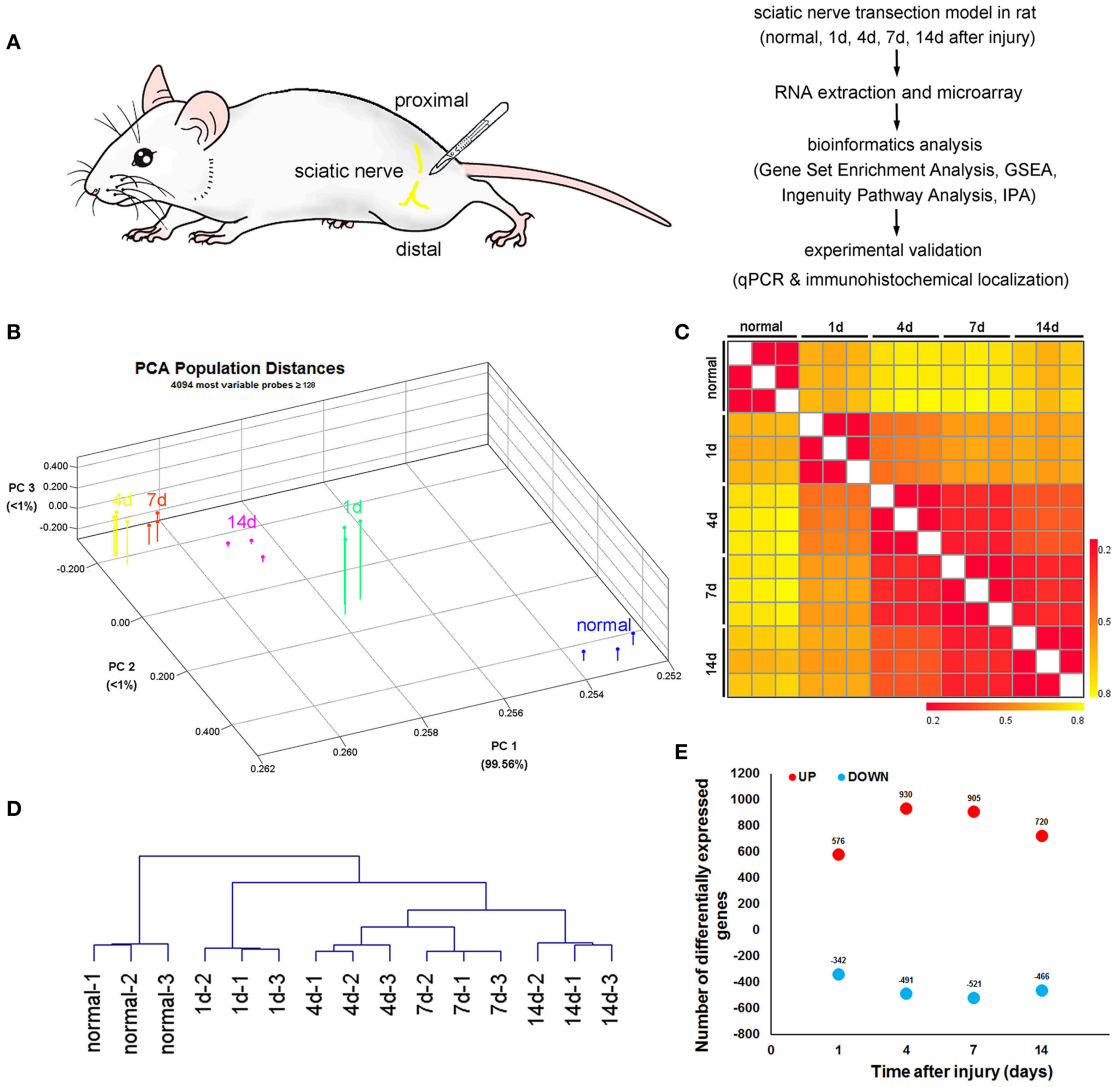
Bird's-eye view of transcriptome changes of the proximal nerve stump after sciatic nerve transection. **(A)** Schematic representation of the experimental paradigm used to uncover the transcriptional program induced by sciatic nerve transection. **(B)** Principal Component Analysis, **(C)** heatmap of Euclidian distances and **(D)** hierarchical clustering between indicated populations, calculated using the 10% of probes with the greatest difference in expression among these subsets and with expression values over 128 for at least one of the subsets. Expression values were log^2^-transformed and row-standardized prior to the analysis. **(E)** Number of probes upregulated (red symbols) or downregulated (blue symbols) by 2-fold or more at the indicated transitions after sciatic nerve transection.

The RNA samples were extracted from proximal nerve stump of rats at different time points (1, 4, 7, and 14 days) following sciatic nerve transection and normal nerve segment in corresponding position as control, followed by the microarray analysis. Several bioinformatics tools of multivariate analysis were used to investigate the relationships among different samples. To further validate the selected genes for immune response and axonal regeneration, both qPCR and histological localization were carried out (Figure 1A).

PCA was applied to map various samples in a 3D space (Figure 1B). The magnitude of transcriptional changes after sciatic nerve transection was better visualized by plotting the cumulative Euclidean distance between various samples (Figure 1C). Unsupervised hierarchical clustering reproduced the known axonal regeneration sequence with striking accuracy (Figure 1D), indicating that there were no hidden surprises in transcriptional relationships along axonal regeneration. The number of transcripts induced or repressed at each time points post-sciatic nerve transection (Figure 1E, Supplementary Table 1).

### Interaction between immune response and axonal regeneration following sciatic nerve transection

**Figure 2:**
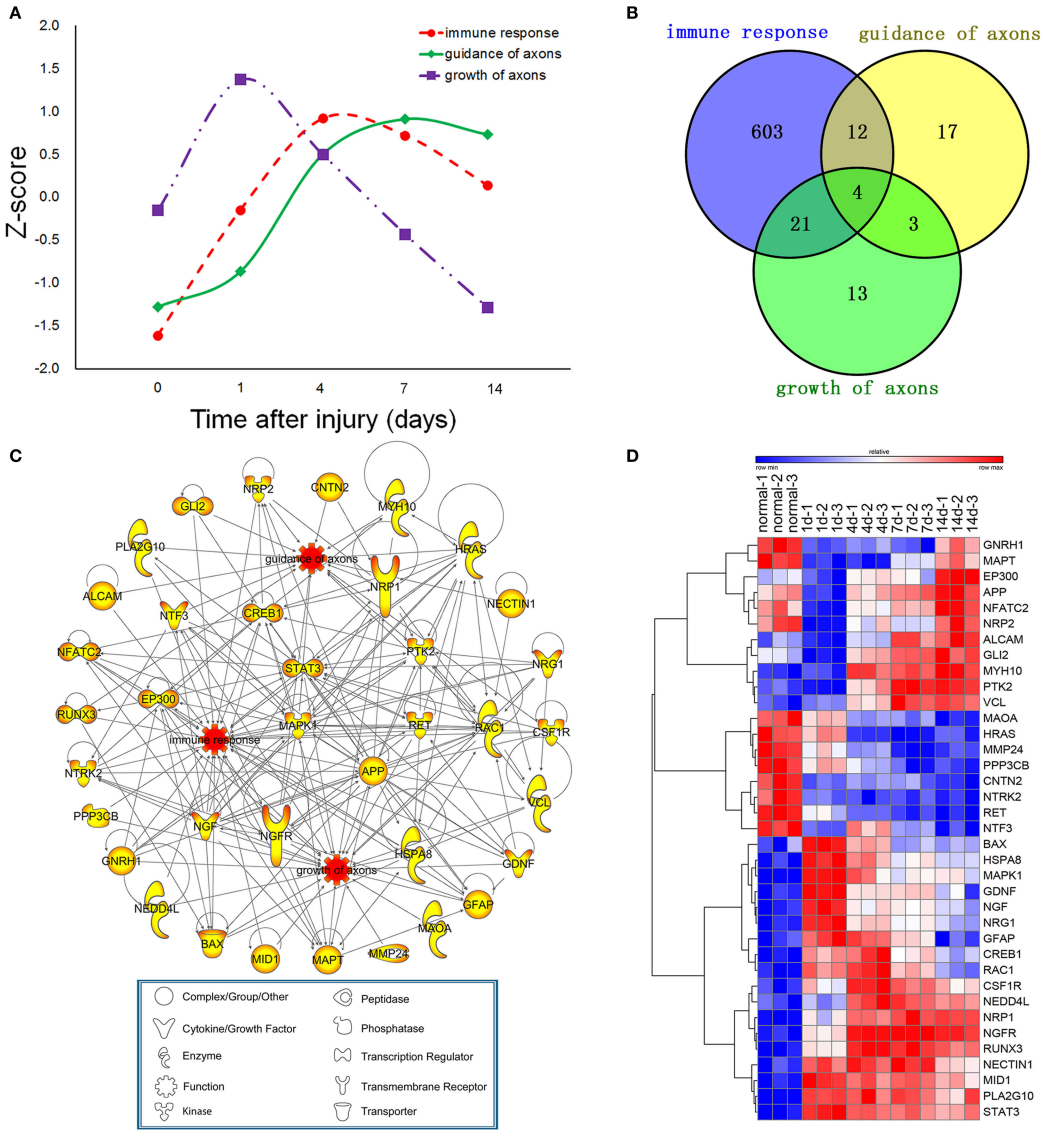
Dynamic changes of differentially expressed genes for guidance of axons, growth of axons and immune response. **(A)** The average expression profiles of differentially expressed genes, which were involved in immune response and axonal regeneration at different time points post-nerve transection. **(B)** Venn diagram showing the number of differentially expressed genes for guidance of axons, growth of axons and immune response. **(C)** Protein- protein interaction (PPI) network for guidance of axons, growth of axons and immune response post-nerve transection. **(D)** Heatmap of dynamic gene expression for guidance of axons, growth of axons and immune response post-nerve transection.

To investigate the interaction between immune response and axonal regeneration following sciatic nerve transection, the average expression profiles of differentially expressed genes (*Z*-score) for immune response, guidance of axons and growth of axons were obtained (Figure 2A). Although the *Z*-score curves showed three different peaks (as 1 day for growth of axons, 4 days for immune response and 7 days for guidance of axons), genes for immune response and guidance of axons showed similar expression trend. The Venn diagram showed that 37 genes involved in all functions of immune response, guidance of axons and growth of axons. According to the IPA database, 37 genes were found involved in the functions of immune response and guidance of axons (12 + 4 = 16 genes), or immune response and growth of axons (21 + 4 = 25 genes), while 4 genes own all functions of immune response, growth of axons and guidance of axons (16 + 25–4 = 37 genes) (Figure 2B). The promotion or inhibition of immune response, guidance of axons and growth of axons determines the outcomes of peripheral nerve regeneration. Therefore, we focused on the differential expression of these related genes and the PPI network linking these genes as well. PPI network was constructed by IPA, which also clearly revealed their molecular types (including enzyme, cytokine, peptidase, phosphatase, transcription regulator, transmembrane receptor, transporter and other types of molecules), functions and interactions (Figure 2C). The dynamic expression level of these 37 genes were showed in the clustered heatmap (Figure 2D) and the data of absolute fold changes were provided in Supplementary Table 1. Based on the screening criteria of multifunctional (significance), differentially expression (color in the nodes) and their novelty (less reported) as well. RAC1, CREB1, NRP1, NRP2, RUNX3, and ALCAM, which were highly correlated 3 biological processes of immune response, guidance of axons and growth of axons, were considered as the hub genes in all nodes of the PPI network investigated here. Therefore, these 6 genes were subjected to further validation (**Figures 5**, **6**). The total gene list of the 3 biological processes described above with the absolute fold changes were also provided in Supplementary Table 4.

### Cascade regulation of transcription regulators in transected nerve stump

**Figure 3:**
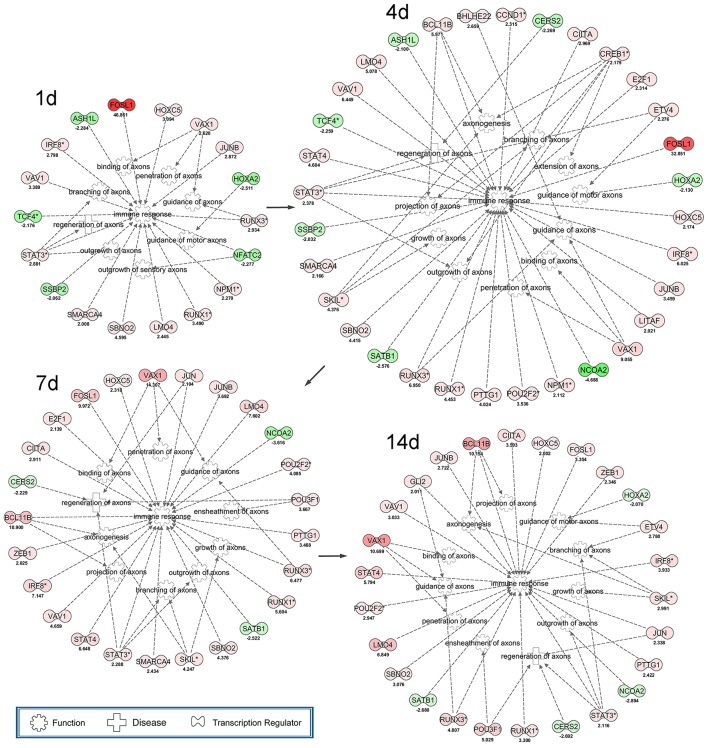
Cascade network of differentially expressed transcription regulators for axonal regeneration and immune response post-sciatic nerve transection. The interaction network of up-regulated (red) transcription factors and down-regulated (green) transcription factors for axonogenesis, regeneration of axons, branching of axons, extension of axons, guidance of axons, guidance of motor axons, projection of axons, growth of axons, outgrowth of axons, penetration of axons, binding of axons, ensheathment of axons and immune response at each time point following nerve transection.

In the present work, the differentially expressed transcription regulators were identified to construct the regulatory networks by the aid of IPA tools (Figure 3). Transcription regulators, including FOSL, HOXC5, IRF8, VAV1, STAT3, SMARCA4, SBNO2, LMO4, RUNX1, NPM1, and JUNB initiated the cascade regulation of immune response from 1 day post-sciatic nerve transection. And VAX1, RUNX3, STAT3 involved in initiating the cascade regulation of binding of axons, guidance of axons and branching of axons, respectively. According to IPA database, the highly expressed (nodes in red color) and multi-functional transcription regulators were considered as the hub genes of all transcription regulators that investigated here. So CREB1 and RUNX3 were selected for further validation (**Figure 5**). At 4 days post-surgery, the regulatory networks was more complex than that at other time points, which suggested a phase transition (4 days) during the whole process of axonal regeneration and immune response following sciatic nerve transection according to IPA database. At 7 days post-surgery, JUN, POU3F1 and ZEB1 were activated for the first time in the cascade of transcription regulators. Among them, POU3F1 initiated the cascade regulation involving ensheathment of axons. While GLI2 was the only new transcription regulators appeared at 14 days post-surgery, which implied the cascade regulation of transcription regulators were totally activated to influence their downstream target genes. Interestingly, once 11 transcription regulators (as FOSL1, HOXC5, IRF8, JUNB, LMO4, RUNX1, RUNX3, SBNO2, STAT3, VAV1, and VAX1) activated at 1 day post-surgery, they had not been disappeared in the cascade regulatory networks of transcription regulators at any other time points.

### qPCR and histological validation of selected genes for both immune response and axonal regeneration following sciatic nerve transection

**Figure 4:**
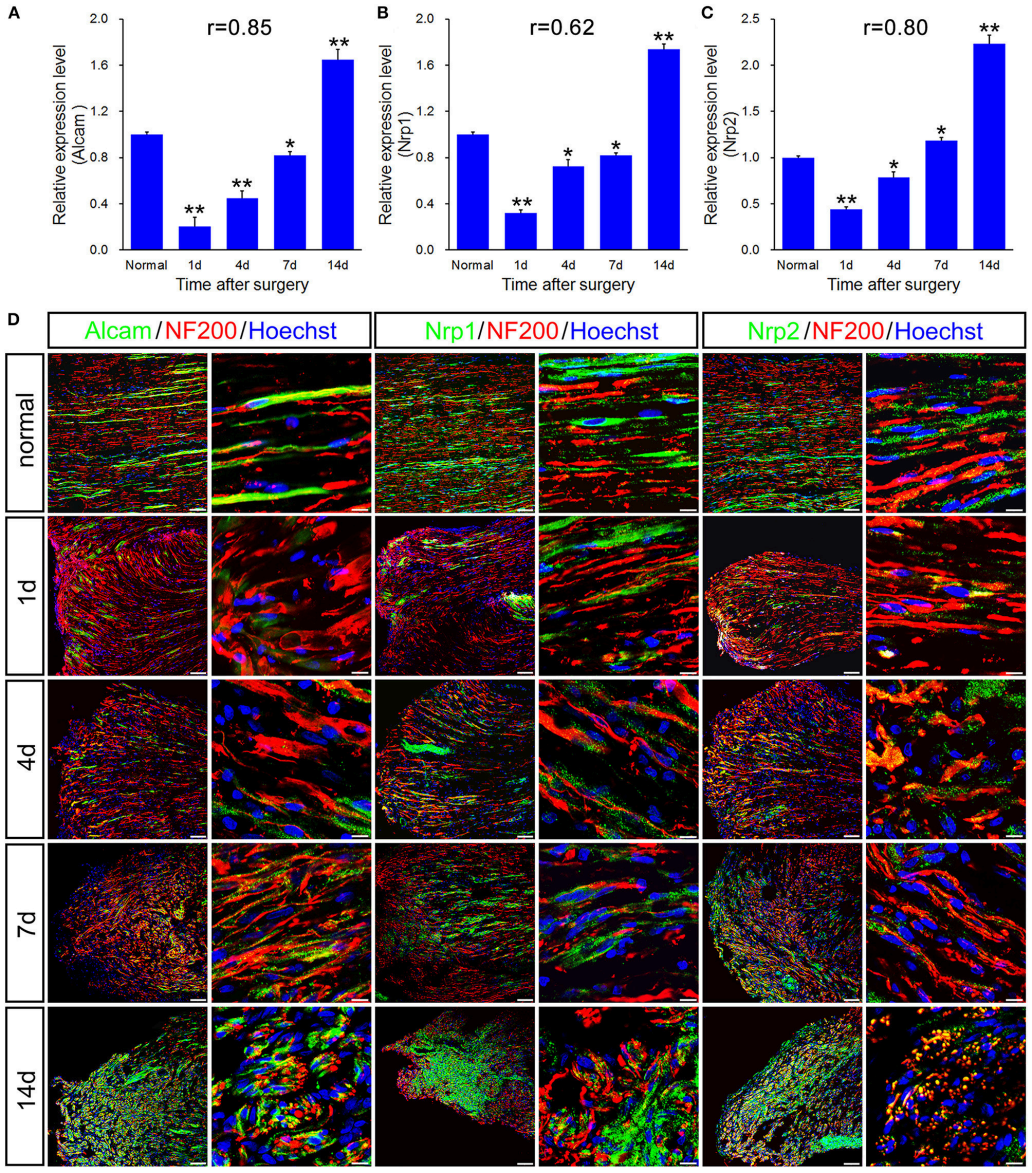
qPCR and histological validation of selected genes for both immune response and axonal regeneration following sciatic nerve transection. **(A–C)** Histograms showing the qPCR validation for relative mRNA expressions of Alcam, Nrp1 and Nrp2. The relative level was normalized to GAPDH. The data, obtained from 3 independent experiments, are expressed as means ± SEM. One-way ANOVA and *post-hoc* Bonferroni *t*-test were used to analyze the data. *P < 0.05, **P < 0.01 vs. the normal control. **(D)** Immunostaining by anti-Alcam, anti-Nrp1 and anti-Nrp2 (green) merged with anti-NF-200 (red) primary antibodies and Hoechst 33342 (blue) obtained at 1, 4, 7, 14 days post-sciatic nerve transection and normal sample of the longitudinal sectioned proximal nerve stump, respectively. Hoechst 33342 marks for nucleus and NF200 markers for nerve fibers. “r” stands for the correlation coefficient. Scale bar, 75 μm (low magnification) and 10 μm (high magnification).

**Figure 5:**
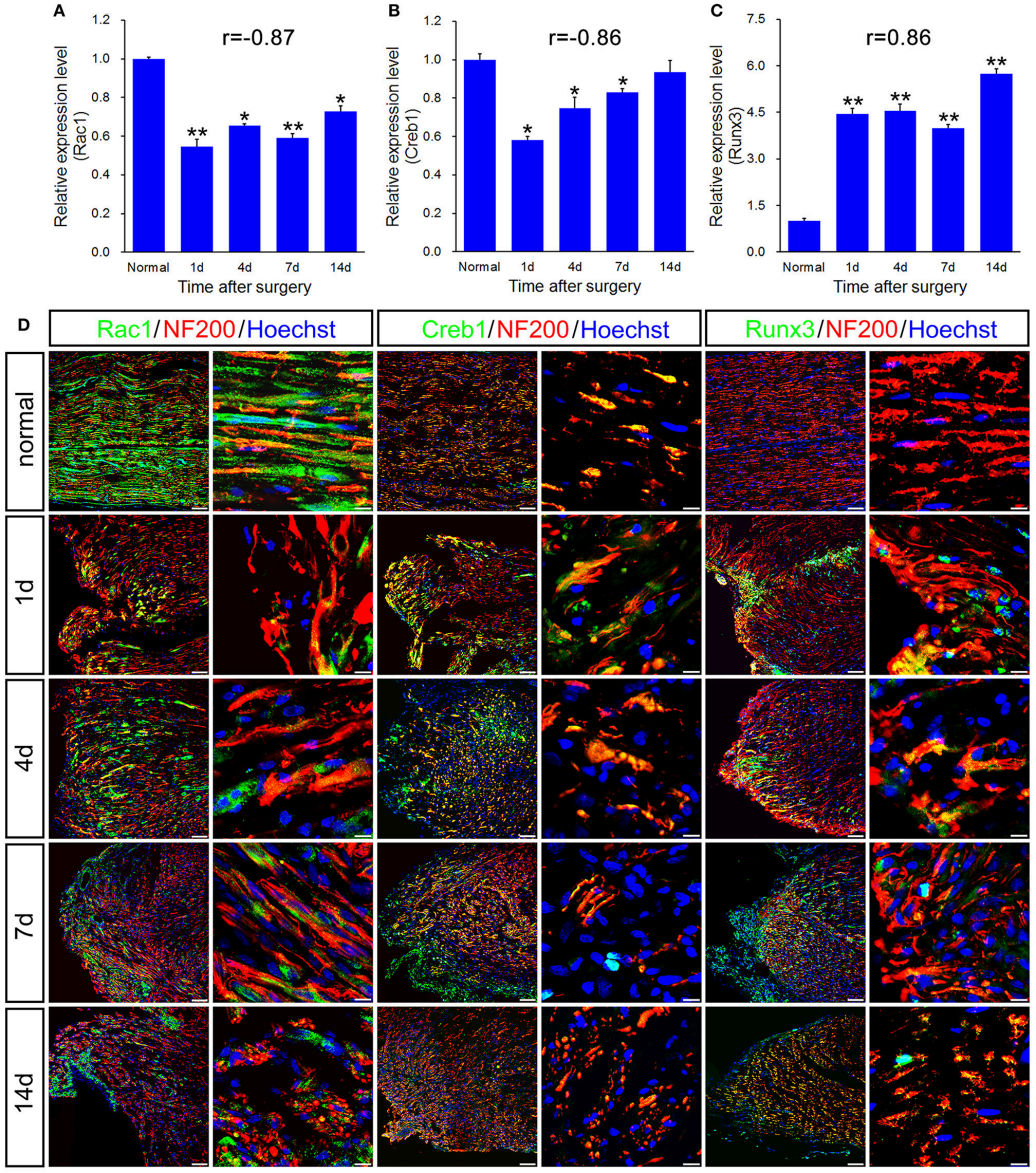
qPCR and histological validation of selected transcription regulators and enzyme for both immune response and axonal regeneration following sciatic nerve transection. **(A–C)** Histograms showing the qPCR validation for relative mRNA expressions of Rac1, Creb1 and Runx3. The relative level was normalized to GAPDH. The data, obtained from 3 independent experiments, are expressed as means ± SEM. One-way ANOVA and *post-hoc* Bonferroni *t*-test were used to analyze the data. *P < 0.05, **P < 0.01 vs. the normal control. **(D)** Immunostaining by anti-Rac1, anti-Creb1 and anti-Runx3 (green) merged with anti-NF-200 (red) primary antibodies and Hoechst 33342 (blue) obtained at 1, 4, 7, 14 days post-sciatic nerve transection and normal sample of the longitudinal sectioned proximal nerve stump, respectively. Hoechst 33342 marks for nucleus and NF200 markers for nerve fibers. “r” stands for the correlation coefficient. Scale bar, 75 μm (low magnification) and 10 μm (high magnification).

qPCR validation for the differential expression of 6 genes including Alcam, Nrp1, Nrp2, Rac1, Creb1, and Runx3, associated with immune response and axonal regeneration (Figures 4A–C, 5A–C). qPCR validation of Alcam, Nrp1, and Nrp2 (Figures 4A–C) showed the similar expression trend which suggested to play similar roles following sciatic nerve transection. The correlation analysis suggested that qRT-PCR data were highly correlated to microarray data (Figures 4A–C, 5C). However, qRT-PCR data of Rac1 and Creb1 were highly negative correlated with the microarray data (Figures 5A,B).

As a subfamily of immunoglobulin receptors with five immunoglobulin-like domains in the extracellular domain, activated leukocyte cell adhesion molecule (ALCAM) which encoded by Alcam gene is also named as cluster of differentiation 166 (CD 166). ALCAM involves in the processes of cell adhesion and migration by the aid of binding to T-cell differentiation antigen CD 6. Mouse Alcam was also reported to involve in guidance and elongation of axons (Chao, 2003; Weiner et al., 2004; Thelen et al., 2012). Nrp1 and Nrp2 encodes two neuropilins (NRP1 and NRP2), which contribute to quite a few signaling pathways of controlling cell migration, as axonal guidance signaling, semaphorin signaling in neurons and VEGF family ligand-receptor interactions. Loss of NRP1 and NRP2 suggested to involve in extension of neurite (Maden et al., 2012).

In principle, GTPase encoded by Rac1 belongs to the RAS superfamily of small GTP-binding proteins, which seems to regulate various cellular events, including the control of the activation of protein kinases, cytoskeletal reorganization and cell growth. It is generally accepted that Rac1 is a positive regulator of axon outgrowth and guidance (Antoine-Bertrand et al., 2016). CREB1 is predicted as the most significant upstream regulator that may be involved in axonal injury (Yasuda et al., 2016). In cancer, Runx3 is commonly deleted or transcriptionally silenced as a tumor suppressor, which also regulates positional variations in axon extension properties without disturbing nerve guidance and branching (Lallemend et al., 2012).

To further validate the molecular variations during sciatic nerve regeneration, triple immunostaining of sciatic nerve longitudinal transection was carried out, which confirm the dynamic expression level of these selected genes following sciatic nerve transection (Figures 4D, 5D). The results showed that these 6 genes were partially localized in the axons, especially in the regenerative axons (yellow areas in the images of 7d or 14d).

### Canonical pathways enrichment analysis for immune response and nervous system signaling following sciatic nerve transection

**Figure 6:**
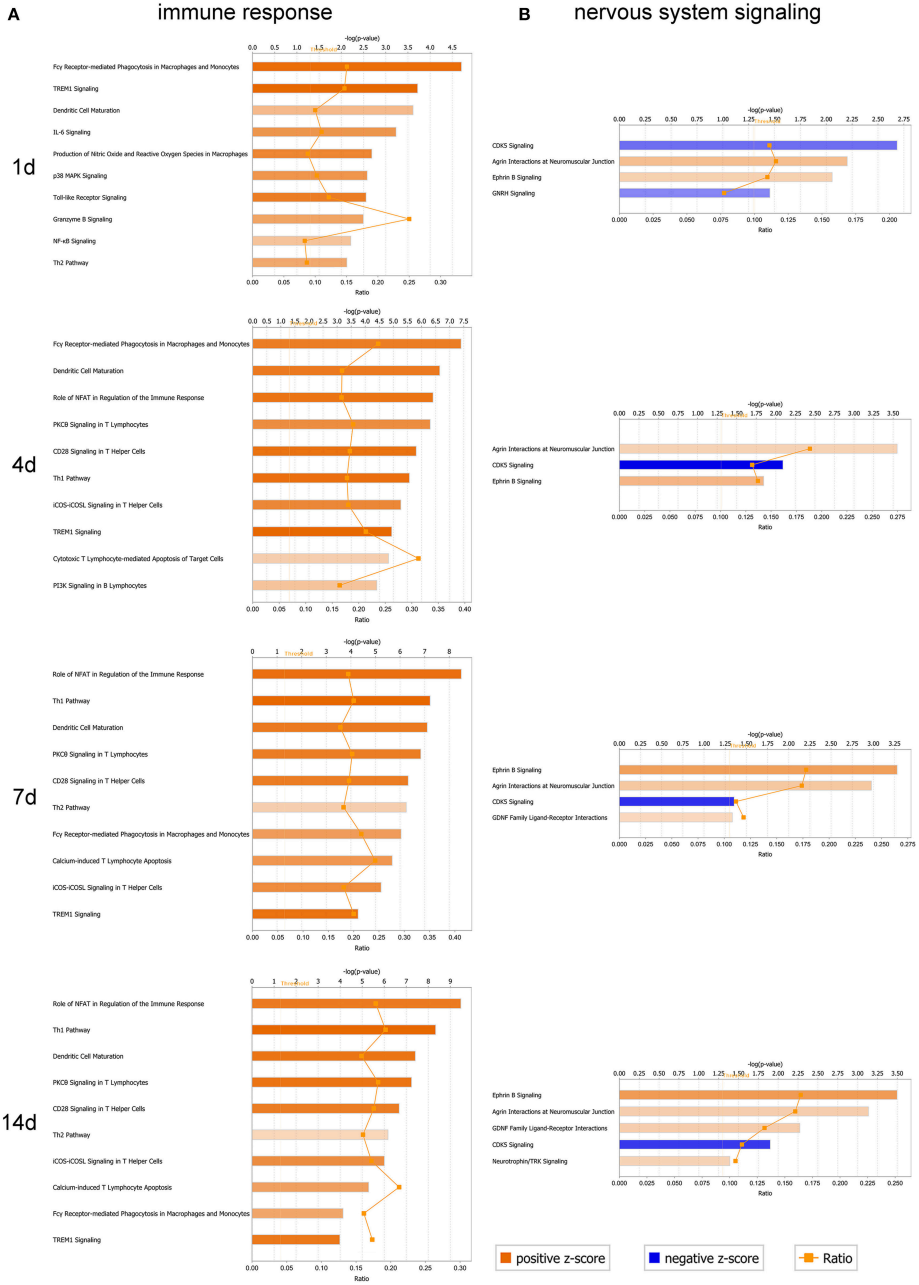
Analysis of canonical pathways enrichment for immune response and nervous system signaling at different time-points post-sciatic nerve transection. **(A)** A selection of significant canonical pathways for immune response, and **(B)** a selection of significant canonical pathways for nervous system signaling, as determined by IPA. Each panel denoted the effect on canonical pathways at particular time-points after sciatic nerve transection. Each bar represents a pathway with significance of enrichment determined using –log (p) value. Orange line represents a ratio (shown on secondary X-axis) of regulated proteins to all proteins in the pathway. The directionality of each pathway was depicted using a pseudo color (orange for activated pathway with positive *Z*-score ≥ 2, and blue for inhibited pathway with negative *Z*-score ≤ −2).

In the present work (Supplementary Table 2), more than 10 canonical pathways for immune response were successively activated (*Z*-core ≥ 2) following sciatic nerve transection, including Fcγ receptor-mediated phagocytosis in macrophages and monocytes, TREM1 signaling, dendritic cell maturation, production of nitric oxide and reactive oxygen species in macrophages, IL-6 signaling, toll-like receptor signaling, p38 MAPK signaling, etc. (Figure 6A). However, the nervous system signaling pathways were enriched in bidirectional status of both activation and inhibition. We only focused on the activated (*Z*-core ≥ 2) pathways including agrin interactions at neuromuscular junction (NMJ), Ephrin B signaling in the early phase (1 and 4 days) and GDNF family ligand-receptor interactions, neurotrophin/TRK signaling in midanaphase (Figure 6B).

Synaptic differentiation is induced by the motor neuron-derived agrin which is essential for the post-synaptic localization of several synapse-specific proteins in the cytoplasm, plasma membrane, and basal lamina. Deposited at the nerve terminal sites which contact with the muscle cells, agrin triggered the development of the NMJ as a signaling factor. A rapid aggregation of acetylcholine receptors (AChR) is induced by agrin in the muscle membrane, primarily through a relocation of preexistent receptors. Synaptic transmission from early stages of NMJ formation is allowed by this agrin-induced clustering of the neurotransmitter receptor (Wu et al., 2010).

While the Eph family plays crucial roles in various biological processes during development as well as in the adult period. As membrane-bound ligands, Ephrins, which are classified into two subclasses based on their mode of membrane anchorage, may activate the Eph receptors. Interactions between Eph receptors and Ephrin ligands are implicated in cell migration, topographic mapping, repulsive axon guidance, and angiogenesis (Cramer and Miko, 2016).

### Transcriptional changes for both immune response and axonal regeneration following sciatic nerve transection

**Figure 7:**
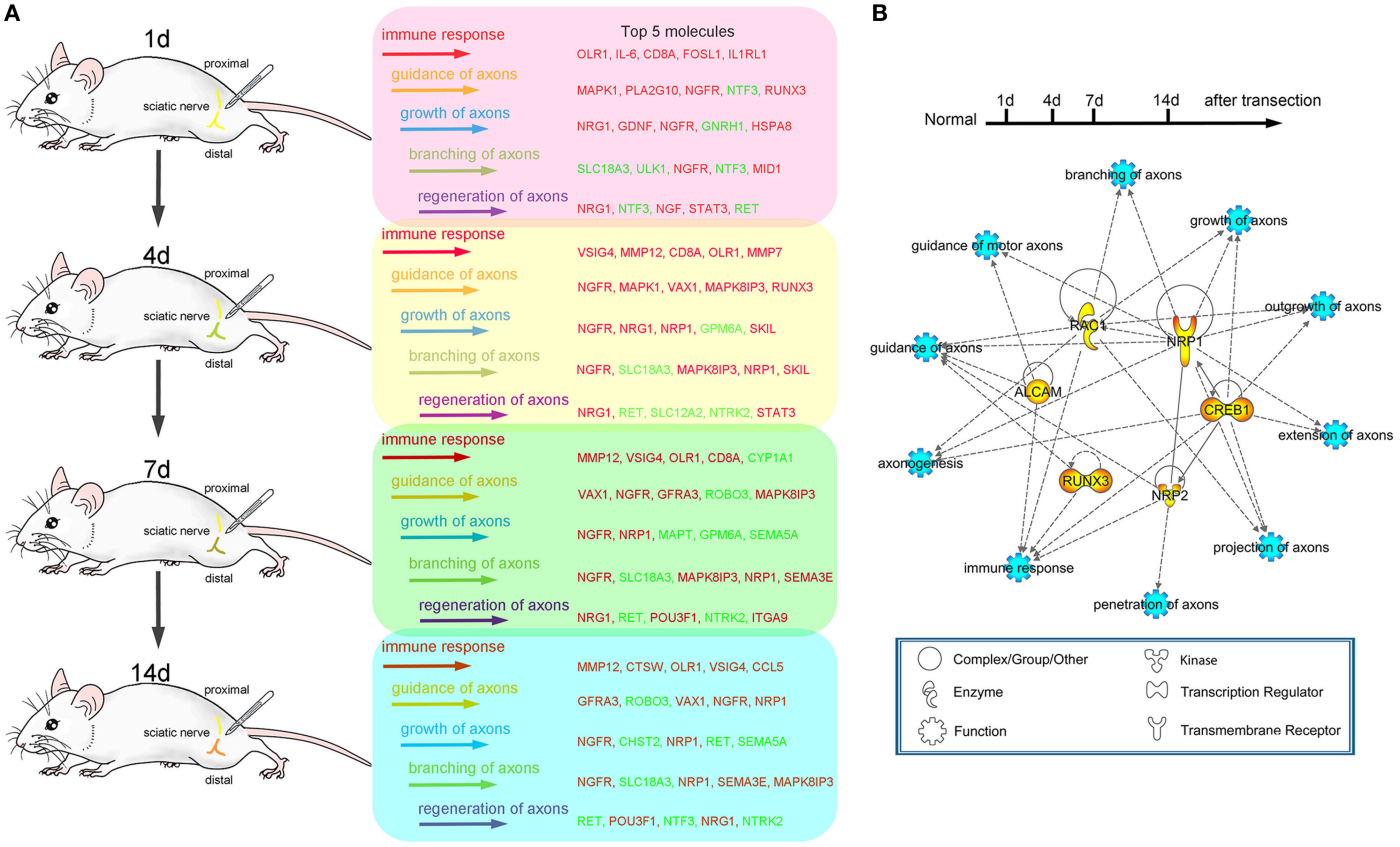
Schematic diagram of the transcriptional changes for both immune response and axonal regeneration following sciatic nerve transection. **(A)** Dynamic changes (top 5) of differentially expressed genes for immune response, guidance of axons, growth of axons, branching of axons and regeneration of axons (red color code for up-regulated genes, green color code for down-regulated genes). **(B)** PPI network of selected genes for axonogenesis, branching of axons, extension of axons, guidance of axons, guidance of motor axons, projection of axons, growth of axons, outgrowth of axons, penetration of axons and immune response.

Schematic diagram (Figure 7A) showed the transcriptional changes for immune response and axonal regeneration (including guidance of axons, growth of axons, branching of axons and regeneration of axons) following sciatic nerve transection. Furthermore, there were several multi-functional genes as the ones we selected for follow-up validation, which generally owned crucial roles after PNI (Figure 7B). This work provides a new way to figure out molecular mechanism of peripheral nerve regeneration.

## Discussion

In the adult mammalian PNS, injured axons maintain the capacity to regenerate spontaneously and provide possibility for functional recovery post-PNI (Abe and Cavalli, 2008). Peripheral nerve regeneration is accompanied by various complicated molecular pathways and cellular events, which are motivated by amount of differentially expressed genes and significantly changed pathways post-PNI. We previously profiled global mRNA expression changes in proximal nerve segments and focused on the dynamic change of the crucial biological processes and also noted the time-dependent expression of key regulatory genes post-sciatic nerve transection (Li et al., 2013). PNI response leads to the upregulation of various genes required for initiating, guiding, and sustaining axonal growth.

To further uncover the molecular mechanism of peripheral nerve regeneration, here we focused on the interaction between immune response and axonal regeneration which associated with specific molecular pathways and interactive networks post-sciatic nerve transection. Our hypothesis is that the genes and molecular pathways for immune response could play crucial roles during axonal regeneration. Therefore, we carried out comprehensive approaches to offer an outline for understanding the specific molecular pathways elaborating in axonal regeneration and attempt to figure out the molecular interaction between immune response and axonal regeneration post-sciatic nerve transection. The results revealed there were over 600 genes and 10 canonical pathways involved in immune response, and that there were only about 60 genes and 5 canonical pathways involved in axonal regeneration. We focused on the genes involved in immune response, guidance of axons and growth of axons based on the IPA database. So amount of differentially altered genes involved in other functions were omitted.

Our data describe that the injury-induced local microenvironment change in proximal nerve stump includes specific molecular pathways and PPI network, which are linked by coordinate actions with the ability of axonal regeneration. Although expressions of the selected six genes were not restricted to the axons, in our present work, we only focused on expression of the selected genes including transcription factors in the terminal of injured axons, to which mRNAs or proteins may transfer from the neuron bodies. The transcription regulators play crucial roles in the regulation of gene expression, which commonly initiate some biological processes or signaling pathways in early phase. An interesting cascade regulatory pattern of transcription regulators has been showed in our present work. Since 11 transcription regulators (as FOSL1, HOXC5, IRF8, JUNB, LMO4, RUNX1, RUNX3, SBNO2, STAT3, VAV1, and VAX1) activated at all-time points post-surgery, all of them may be the key transcription regulators during the process of neural regeneration. Four days is considered as the initiated time point of ensheathment of axons in molecular-level analysis. An alteration in the populations of mRNAs localized to axons is activated by axotomy, demonstrating a dynamic regulation of the axonal transport machinery. In response to axonal stimuli, axonal mRNA localization and transport are regulated to achieve precise variations of axonal RNA content (Yoo et al., 2010). Compared to the work we published previously (Li et al., 2013, 2015), we specifically focused on the biological processes including immune response, guidance of axons and growth of axons in the present work instead of extensive research with systematic description. The 6 genes including Alcam, Nrp1, Nrp2, Rac1, Creb1, and Runx3 were firstly identified in rat models suffering sciatic nerve transection, which were highly correlated with immune response and axonal regeneration. The investigated gene transcripts (Figures 4, 5) were found partially localized in the axons, especially in the regenerative axons (yellow areas in the images of 7 or 14 days). Previously, Alcam mRNA was found in axonal growth cones of retinal ganglion cells (RGCs) *in vivo* and *in vitro*. The translation of Alcam mRNA may occur in RGC growth cones which separated from their soma (Thelen et al., 2012), which strongly supported our results.

As an important approach to analyze coordinate expression changes at a pathway level rather than focusing on a single gene, enrichment analysis of canonical pathways was carried out using IPA to further explore the regulation of immune response and axonal regeneration (Supplementary Table 2). The nodes of the network contain many previously identified regeneration-associated genes. Our results showed that the injury-induced local microenvironment change in proximal nerve stump includes specific molecular pathways and PPI networks. Furthermore, mechanistic-based treatments will also be developed based on our results, which can be used as tool for screening drugs or small molecular compounds that may enhance axonal regeneration (Chandran et al., 2016). The PNI can activate both the innate and the acquired immune responses. Of note, the success or failure of the regeneration process has been interfered by the interact and overlap mechanisms of nervous and immune system after PNI (Gaudet et al., 2011; Rotshenker, 2011; Shastri et al., 2013; Xanthos and Sandkuhler, 2014).

Our work provide a new way to figure out molecular mechanism of peripheral nerve regeneration and should provide a valuable resource to figure out the molecular processes which outline neural injury-induced micro-environmental variation to discover novel therapeutic targets for axonal regeneration.

## Author contributions

CX, XG, and SC conceived and designed the experiments. QG, HZ, and HW performed the experiments. SW, PZ, and ZS analyzed the data. SL and HZ contributed reagents, materials and analysis tools. QG, HZ, and CX prepared and revised the manuscript. The final manuscript has been read and approved by all authors.

### Conflict of interest statement

The authors declare that the research was conducted in the absence of any commercial or financial relationships that could be construed as a potential conflict of interest.
